# Biomass carbon removal can help sustainable aviation fuels achieve on-time arrival

**DOI:** 10.1016/j.isci.2026.115956

**Published:** 2026-05-22

**Authors:** Matthew Langholtz, Charlotte Levy, John Field, Daniel L. Sanchez, Pete Christensen, Lawrence Murdoch, Daniel de la Torre Ugarte, Oluwafemi Oyedeji, Ryan Jacobson, Ning Zeng, Emily A. Heaton, Charles Forsberg, William Joe Sagues

**Affiliations:** 1 Oak Ridge National Laboratory, Oak Ridge, TN, USA; 2 Carbon180, Washington, DC, USA; 3 University of California, Berkeley, Berkeley, CA, USA; 4 Loamist Co., Berkeley, CA, USA; 5 Clemson University, Clemson, SC, USA; 6 University of Maryland, College Park, MD, USA; 7 University of Illinois Urbana-Champaign, Champaign, IL, USA; 8 Massachusetts Institute of Technology, Cambridge, MA, USA; 9 North Carolina State University, Raleigh, NC, USA

**Keywords:** environmental science, biomass, bioresources, bioenvironmental engineering

## Abstract

Biofuels, including sustainable aviation and marine fuels, and biomass carbon removal and storage (BiCRS) are often viewed as potentially competing pathways for advancing climate and energy goals. Their comparative economic, environmental, and temporal advantages remain debated. Rather than identifying a “best-use” for biomass, we show that the relative economic advantages of BiCRS versus biofuels exist along a continuum shaped by energy- and decarbonization-focused market conditions. These pathways need not be adversarial: BiCRS can enable, rather than displace, future biofuel deployment. While the lignocellulosic biofuel sector continues to face barriers associated with underdeveloped supply chains and technologies that have not yet been commercialized at scale, emerging BiCRS approaches are comparatively feedstock-flexible, rapidly deployable, and responsive to carbon removal markets. Early BiCRS deployment can help establish reliable biomass supply chains, reducing investment risk for future lignocellulosic biorefineries. By easing initial supply chain constraints, BiCRS can serve as a practical stepping stone toward meeting both near-term carbon removal needs and long-term sustainable fuel objectives under uncertain future market and policy conditions.

## Biofuels and BiCRS opportunities and challenges

Near the turn of the century, the term “peak oil” was widely interpreted as a looming supply-constrained crisis in which diminishing petroleum resources and rising demand could lead to economic disruption.^1^ However, the rapid expansion of unconventional oil production, particularly through hydraulic fracturing in the United States, together with accelerated deployment of renewable energy technologies and efficiency improvements, has shifted the dominant narrative from peak supply to peak demand.^2^^,^^3^ These conflicting interpretations of “peak oil” imply fundamentally different market trajectories: one characterized by fuel scarcity and high prices, the other by long-term fuel abundance and downward price pressure. For capital-intensive biofuel production infrastructure with multi-decadal lifetimes, this divergence exemplifies how uncertainty in long-run fuel price expectations can complicate investment decisions and delay deployment.

Climate-mitigation goals face similarly deep uncertainties. Recent COP deliberations, including the COP28 global stocktake and COP30, highlight persistent uncertainty in the pace and mechanisms of global emissions reductions, contributing to continued ambiguity in long-term policy signals needed to guide investment in low-carbon technologies.^4^^,^^5^ Biomass resources are widely recognized as having strategic value across this uncertain landscape—both as feedstocks for renewable liquid fuels (e.g., sustainable aviation fuel [SAF], renewable diesel, etc.) and as inputs to biomass-based carbon removal pathways.^6^ Yet, the optimal allocation of biomass depends strongly on future market and policy conditions, which are unknown. These uncertainties raise critical questions for bioeconomy development: how should biomass be deployed to best support evolving energy and carbon-management objectives? And how a resilient bioeconomy can be designed to perform under potentially divergent energy- and climate-focused futures?

Biofuels represent a leading alternative to conventional fuels in transportation sectors that are difficult to electrify, including aviation and maritime shipping. Current research suggests that biofuel production and use is the most technologically and economically mature renewable option for these applications, offering operational viability, potential environmental benefits, and support for regional economic development.^7^^,^^8^^,^^9^^,^^10^ Among the co-benefits of bioenergy are uptake of atmospheric carbon dioxide (CO_2_), improved water quality and reduced field emissions associated with reduced fertilizer inputs of perennial crops, and opportunities to improve soil health, if implemented within environmental guardrails.^11^^,^^12^ However, the environmental outcomes of bioenergy systems are pathway- and context-dependent. Life cycle assessments indicate strong performance for residues and sustainably managed feedstocks, but net climate benefits may be reduced where indirect market effects or intensive inputs increase life cycle emissions, water quality issues, or other negative externalities. Similarly, while perennial systems can enhance soil carbon, biodiversity, and certain ecosystem services relative to annual cropping systems, the literature highlights both synergies and trade-offs associated with their deployment. For example, perennial bioenergy crops may also increase water use or influence local water availability depending on crop type, regional climate, and land management practices.^8^^,^^9^^,^^13^^,^^14^^,^^15^^,^^16^

Scaling up the bioeconomy for SAF and co-products presents deployment challenges. Though operationally feasible, bioeconomy growth is hindered by underdeveloped biomass supply chains.^17^ Such growth faces a chicken-and-egg problem, where a lack of established biomass supply chains poses a risk to large-scale biorefinery deployment, but supply chains develop slowly without concentrated local market demand. Robust SAF and marine fuel markets need both buyers and sellers to be successful, but it is difficult to attract one without the other. Rapid 2025 policy shifts, including the reorientation toward domestic energy expansion and streamlined approvals, underscore both opportunities and uncertainties for large-scale biofuels investment. Considering the existing uncertainties related to market dynamics, policy stability, and technological progress, what strategies can be employed to mitigate risks and achieve energy, environmental, and economic objectives associated with biofuels and the bioeconomy?

One strategy to develop biofuel supply chains amid uncertainties in market dynamics, policy frameworks, and technological advancements is to leverage alternative, large-scale, and low-cost biomass markets. Once established, those supply chains would demonstrate the viability of novel biomass sources and crops, generate information on the cost and reliability of producing such feedstocks, and provide a base for growth if and when other end-uses (such as advanced biofuel production) emerge. This approach facilitates the establishment of supply chains capable of supporting multiple end uses and could help reduce the costs of biomass feedstock, one of the largest portions of the overall supply chain cost.^18^^,^^19^^,^^20^

One promising application with significant growth potential is biomass carbon removal and storage (BiCRS). BiCRS projects seek to manipulate biomass so that the carbon it contains—originally captured from the atmosphere during plant growth—is stored rather than allowed to decompose and flow back to the atmosphere.^21^ Different approaches to BiCRS include burying biomass under conditions that prevent microbial decomposition, converting biomass into recalcitrant biochar for long-term storage in soils or through dedicated burial, or converting it into bio-oil that can be injected into geological formations for permanent sequestration.^22^ Microsoft and the Frontier advance voluntary market commitment—which is supported by Stripe, Alphabet, Meta, Shopify, and other corporate buyers—are creating significant industry demand for carbon removal via BiCRS and related approaches.^23^ From 2020 to 2024, BiCRS accounted for more than 80% of delivered CO_2_ removal (CDR), but while receiving less than 3% of CDR investments.^24^

## Bioenergy vs. BiCRS: Competing strategies?

BiCRS and bioenergy strategies are summarized as follows:

1. BiCRS: focused on carbon management, BiCRS pathways remove CO_2_ from the atmosphere and store it underground or in long-lived products (e.g., biochar, burial, building materials). BiCRS offers significant, cost-effective opportunities for negative emissions, potentially removing 800 million tons (Mt) of CO_2_ annually in the US.^21^ Some of these approaches are small-scale and modular (a few hundred thousand tons of feedstock per year, or less), requiring low capital costs and minimal upfront investment,^25^^,^^26^ which enables distributed deployment across a wide range of geographies. To ensure credibility and scalability of carbon removals, monitoring, reporting, and verification (MRV) systems must be strengthened—particularly for small-scale BiCRS technologies such as biomass burial and biochar application. As with biofuels, the net climate benefits of BiCRS depend on feedstock sourcing, upstream supply-chain emissions, and long-term storage permanence, and require careful accounting of potential ecosystem impacts or other externalities. BiCRS projects are projected to provide 20 Mt per year of high-durability carbon removal by 2030, based on developer scale-up plans.^27^
2. Bioenergy (with emphasis on biofuels for hard-to electrify sectors, e.g., SAF and marine fuels): focused on fuel production and net emissions avoidance, biofuels provide renewable energy and enhance energy security. The US Air Force, for example, spends nearly $7 billion annually on jet fuel and has certified its fleet for a 50/50 blend of certain synthetic aviation turbine fuels. However, SAF production is highly capital-intensive, relying on complex infrastructure, large-scale (∼700,000 tonnes per year^28^) centralized facilities, and robust, large-volume supply chains. Its economic viability heavily depends on economies of scale, creating substantial barriers to entry for new producers.

Key attributes to compare SAF, marine, or other biofuels with BiCRS include (1) volume of products, e.g., fuels and coproducts, (2) carbon abatement potential, i.e., tons of CO_2_ sequestered or avoided, net of supply-chain emissions, and (3) carbon abatement cost, i.e., $ per ton CO_2_ abated, calculated as marginal cost of the biomass option divided by net tons of CO_2_ abated (i.e., Equation S3). These attributes are used in following sections to explore the comparative advantages of biofuels (e.g., SAF) and BiCRS for the US and for global climate commitments.

## Market drivers and incentives globally and in the US

The potential magnitude of a mature biofuels sector is well illustrated by on-going initiatives. The International Air Transport Association has set an ambitious goal to achieve net-zero aviation emissions by 2050, aiming for the annual production of 450 billion liters of SAFs, a figure approximately 350 times greater than the current global production levels.^29^ Concurrently, the European Union and other organizations have established targets to mitigate shipping emissions through the adoption of marine biofuels and alternative strategies. For example, the European Union’s “Fit for 55” policy framework includes the ReFuelEU Aviation and FuelEU Maritime regulations, which mandate increasing use of sustainable fuels in aviation and set progressive carbon-intensity reduction targets for marine fuels through 2050.^30^

**Figure 1:**
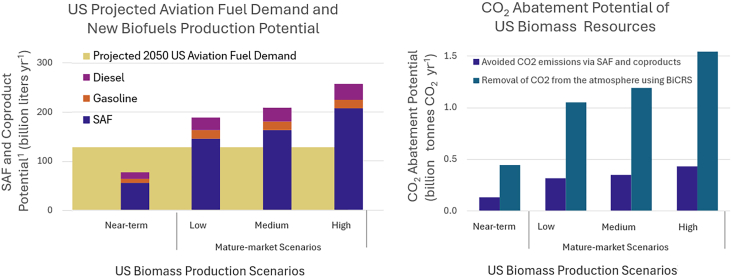
Illustrative allocation of US biomass resources between SAF and co-product production (left) and CO_2_ abatement potential of SAF vs. BiCRS (right) US biomass resources are more than adequate to meet SAF production goals but can provide more CO_2_ abatement via BiCRS than SAF. Based on US biomass availability under near-term and mature-market conditions (mature-market scenarios are defined in Table 1.2 of DOE [2024].^8^ These scenarios represent estimates of biomass supply-side production capacity under sustained price signals, subject to specified economic, environmental, and land-use constraints, while continuing to meet conventional demands for food, feed, fiber, and exports. They are not forecasts, but illustrative assessments of potential supply under assumed market conditions, and are policy- and end-use agnostic. Across the three mature-market scenarios, assumptions for waste and forestry resources are held constant, while agricultural resource availability varies according to the assumptions described in Table 5.1 and illustrated in Figure ES-1 of the billion-ton Report.), excluding current US bioenergy and biofuels uses (DOE 2024).^8^ Conversion assumptions for SAF and co-products are derived from National Renewable Energy Laboratory’s “Biofuel Production and Greenhouse Gas Reduction Potential” (Brown and Tao, 2023)^31^ and are provided in the SI.

**Figure 2:**
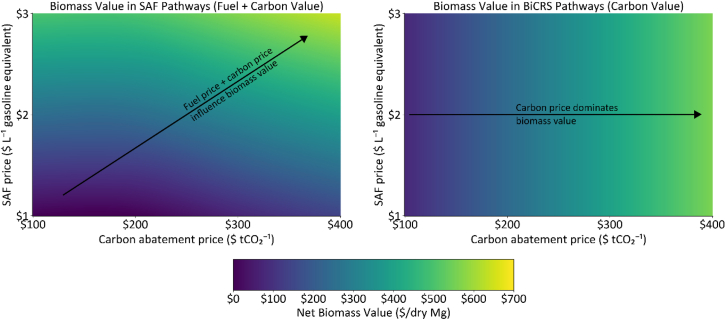
Illustrative biomass valuation under representative SAF and BiCRS conversion assumptions across a range of fuel and carbon abatement prices Biomass valuation in SAF pathways increases with both fuel price and carbon price due to the combined value of fuel production and associated carbon abatement, whereas biomass valuation in BiCRS pathways depends primarily on carbon price. Representative assumptions for SAF, 250 L gasoline equivalent per ton biomass, $1.00 per liter SAF gasoline equivalent break-even cost, 0.7 tons CO_2_ abated per ton of biomass; and for BiCRS, 1.6 tons CO_2_ per ton of biomass, $50/t CO_2_ carbon abatement cost. Calculation equations are provided in SI page 9.

The US provides a useful case study because its large and well-characterized biomass resource base is paired with a policy environment that includes national energy policies as well as strong state-level initiatives and private-sector investment driving the development of climate solutions. This analysis adopts the US as the primary spatial boundary for quantitative illustrations (Figures 1 and 2), drawing on biomass availability estimates from the 2023 billion-ton report.^8^ Those estimates explicitly incorporate environmental and economic sustainability constraints, including protection of food, feed, and fiber markets, maintenance of soil carbon and water quality, and limits on land-use change, as detailed extensively in the underlying report.^8^^,^^11^ The scenarios referenced here reflect supply-side production potential under mature-market conditions and are policy- and end-use-agnostic, rather than forecasts of realized deployment. Market illustrations in Figure 2 represent generalized region-agnostic fuel and carbon price conditions rather than specific policy commitments or regulatory trajectories.

From a supply perspective, the US has more than enough biomass production capacity to meet its projected demand for aviation fuels. The US can triple the scale of its current bioeconomy to produce approximately 1.4 billion tons of biomass per year, within environmental and economic constraints, by increasing its use of existing agricultural and forestry residues and wastes, and by cultivating new purpose-grown energy crops.^8^ Such an increase in biomass supply could produce over 160 billion liters per year, more than the projected US jet fuel demand of ∼130 billion liters per year in 2050^32^ (Figure 1). If good practices^11^ are followed, significant energy, economic, and environmental benefits can be achieved^33^ and potential negative environmental impacts can be avoided.^8^^,^^9^ For instance, producing perennial energy crops on low-yielding or abandoned agricultural land can contribute to environmental and carbon-management goals while minimizing impacts to existing agricultural production.^14^^,^^21^^,^^34^ Low-input, resilient perennial crops can diversify farm income with stable, predictable yields,^35^^,^^36^ which can be an asset in the face of commodity price uncertainty and extreme weather events. Markets for lower-value wood can facilitate wildfire risk mitigation through increased forest thinnings.^37^

## Evaluating the availability of biomass against future demands

Figure 1 illustrates a mutually exclusive allocation (for illustration) of US biomass resources between SAF production and BiCRS. If used for SAF, biomass resources in the US can produce upwards of 200 billion liters per year of SAF and coproducts while abating ∼0.3 billion tons per year of CO_2_. If instead deployed for biomass burial as an example BiCRS pathway, no fuels are produced, but upwards of 1 billion tons per year of CO_2_ could be sequestered (Figure 1, right). Due to high carbon removal efficiency and very low conversion costs, the BiCRS example can achieve carbon abatement costs (CACs) of ∼$10–$200 per ton CO_2_,^21^^,^^26^^,^^38^^,^^39^ lower than the ∼$400–$1,000 per ton CO_2_ CAC estimated for SAF pathways.^40^ Under current conditions and in the absence of government incentives or fuel blending mandates, BiCRS, therefore, represents a far more cost-effective and scalable approach to carbon management than SAF or other biofuel-based pathways. Adding gaseous CO_2_ capture and storage to SAF production refineries can increase the quantity of CO_2_ abatement potential of SAF, but with additional costs. Together, SAF and BiCRS span a continuum of energy and carbon management strategies: SAF prioritizes fuels production and associated abatement, whereas BiCRS offers superior performance in durable, net-negative carbon removal. Both pathways may be useful for reaching future goals of fuel production and emissions reductions, although future policy priorities, market conditions, and price signals are required to realize those goals are uncertain.

In addition to the illustration in Figure 1, the potential tension between bioenergy and BiCRS approaches is illustrated in the Roads to Removal Report.^21^ Figure 6.2 of the report compares a BiCRS-optimized decarbonization scenario with an SAF-focused decarbonization scenario that additionally provides 66 billion liters per year of SAF. The SAF scenario decreased US CO_2_ abatement potential from 877 to 712 million tons per year, and increased carbon abatement prices, relative to a BiCRS-optimized scenario. In addition to the quantitative differences, SAF and BiCRS research communities can be compartmentalized. Anecdotally, SAF conference participants are often unfamiliar with, or even skeptical of, BiCRS. However, in contrast to this skepticism, major corporate buyers—including Microsoft, Google, BCG, and JPMorgan—have already contracted more than 3 million tons of biomass-based carbon removal since 2022, including 1.6 million tons in just the first half of 2025, demonstrating that BiCRS is being deployed at substantial and rapidly growing scale.^41^

## Relative economic value of SAF and BiCRS pathways across market conditions

Intuitively, the relative per-ton economic value of biomass under SAF and BiCRS pathways depends on pathway-specific conversion efficiencies and costs, as well as fuel and carbon price assumptions. However, a collective, quantitative understanding of the comparative advantages of these two approaches remains elusive. To elucidate the comparative advantages of SAF and BiCRS pathways under a continuum of energy- and carbon-focused market conditions, here we illustrate the per-ton biomass economic value, net of conversion costs. These are calculated as the value of fuels and CO_2_ avoidance for SAF (Equations S1 and S2), and the value of CO_2_ removal for BiCRS (Equation S2) under a range of biofuels and carbon abatement prices. In practice, results will vary by feedstock- and pathway-specific technoeconomic assumptions, policies, and potential valuation of other products or externalities. To simplify the analysis, biomass values are calculated under representative SAF and BiCRS conversion efficiencies and costs (SI page 9). Results under a range of fuel and price scenarios are shown in Figure 2. Rather than identifying a single optimal use, this analysis illustrates how the relative value derived from cellulosic biomass under SAF and BiCRS pathways shifts across plausible fuel and carbon price regimes under representative conversion assumptions.

The per-ton biomass valuations in Figure 2 illustrate that, under representative conversion yields and costs for SAF and BiCRS, where market signals are high for fuels, but are low for carbon abatement, SAF is economically advantaged, and vice-versa for the BiCRS scenario. This aspect of the potential relative advantage of biofuels vs. BiCRS to achieve potentially complementary, though different, objectives of renewable fuels and carbon abatement needs to be considered when exploring strategies to address complex geo-scale societal objectives.

The contrast between the two sections in Figure 2 highlights that the value of biomass depends on fundamentally different market drivers for SAF and BiCRS. For SAF, biomass value increases primarily with higher fuel prices; for example, as the fuel price approaches $2.50 per liter, the biomass value rises sharply into the green region. Variation in carbon price, however, has only a modest effect because each liter of SAF delivers relatively limited emissions abatement. In contrast, the BiCRS panel shows that biomass value is insensitive to fuel price and depends on the carbon price. Although the shift from white to green is subtle at lower carbon prices, biomass value increases steadily with higher carbon prices and grows substantially once prices exceed roughly $200 per ton CO_2_, reflecting the high carbon removal per ton of biomass stored. Indeed, carbon removal credits are currently being routinely sold at above this threshold.^42^ Conversely, SAF is not achieving equivalent pricing—current estimates place SAF costs at approximately $9.40–$10.96 per gallon (about $2.48–$2.91 per liter), indicating that it is not yet competitive with conventional jet fuel without strong policy or premium pricing.^43^ Although SAF production costs are expected to decrease over time, achieving such cost reductions will depend on building large, centralized production facilities and developing robust, high-volume biomass supply chains capable of supporting sustained commercial operation. Together, these results demonstrate that SAF and BiCRS currently reward biomass under very different economic conditions.

Unknown future technologies, economic conditions, policies, and market prices will all influence the scenario assumptions illustrated in Figure 2, making definitive predictions about the best uses of biomass impossible. For example, lower conventional oil prices can put downward pressure on SAF prices, which can make biomass valuation less profitable from a fuels perspective (Equation S1). At the same time, lower SAF prices increase the carbon abatement costs of SAF (Equation S3), which reduces biomass valuation from a carbon abatement perspective (Equation S2). Together, these effects decrease the combined biomass valuation in the SAF pathway (sum of Equations S1 and S2). However, proposed working generalizations from this framework are summarized in Table 1. Future market conditions that prioritize energy are more likely to favor biofuels pathways, whereas conditions that prioritize carbon abatement are more likely to favor BiCRS. In scenarios where markets place high value on both SAF and carbon abatement, the preferred use of biomass remains uncertain, given unresolved technology performance, policy design, and market evolution. Moreover, the deployment for either SAF or BiCRS at scale is constrained by underdeveloped supply chains,^40^^,^^44^^,^^45^ motivating the supply chain development strategies discussed below.

**Table 1:** Relative advantages of BiCRS and SAF under a generalized matrix of energy and carbon abatement demand scenarios

		Biofuel value	
		Low	High
Carbon abatement value	Low	Low demand for BiCRS or Biofuels	Biofuels advantaged
	High	BiCRS advantaged	Optimal uses will be determined by TEA, LCA, policy, and economic conditions

While BiCRS and bioenergy have distinct near-term objectives, they can also function as sequentially synergistic components of a unified bioeconomy. Early deployment of BiCRS projects—including biomass burial, biochar application, and bio-oil injection (in this study, bio-oil refers to the liquid intermediate produced from fast pyrolysis of lignocellulosic biomass, composed mainly of oxygenated hydrocarbons and water)—can establish the physical and institutional foundations required for large-scale biofuel expansion. The steady biomass demand created by BiCRS helps justify investments in feedstock aggregation, storage, and transportation infrastructure, driving down delivered-biomass costs through improved logistics, equipment utilization, and supply reliability. These early investments reduce one of the largest long-term cost barriers facing SAF producers and strengthen grower participation by providing stable, diversified markets for residues and energy crops. Importantly, these synergies are temporal rather than simultaneous; BiCRS creates enabling conditions for SAF only after sufficient learning, infrastructure build-out, and policy support accumulate. At any given moment, the two pathways do not exhibit strong operational synergy, and the degree to which BiCRS lowers future SAF costs depends heavily on dynamic market forces and incentives.

It is important to note that certain lignocellulosic supply chains are already well developed for traditional bioenergy uses, particularly for heat and power generation. The wood pellet industry in the southeastern US, for example, has approached production and export levels on the order of 10 million tons per year, largely supplying European renewable energy markets.^46^ This sector demonstrates that large-scale, long-distance biomass logistics are technically and economically feasible, and it provides a valuable model of supply-chain maturity. However, this infrastructure primarily draws on forestry resources rather than agricultural residues, leaving a significant gap in established supply systems for agricultural lignocellulosics such as corn stover, wheat straw, and cotton gin trash.

While agricultural residues could also serve traditional bioenergy markets—for example, by replacing natural gas with corn stover to provide process heat at existing corn ethanol mills—such applications have seen limited commercial progress. Although this substitution could reduce the carbon intensity of ethanol production, the lack of consistent demand and supportive policy frameworks has slowed broader adoption. In contrast, BiCRS provides a growing market signal for these underutilized agricultural residues, creating new incentives for collection and processing.

Because BiCRS technologies generally operate at smaller scales and rely on modular systems with lower capital intensity than boiler-turbogenerator configurations for heat and power production, they can expand more rapidly and at lower cost.^21^^,^^26^^,^^38^^,^^39^^,^^40^ Some BiCRS pathways are also inherently simple from an engineering perspective. For example, biomass burial approaches rely primarily on drying and isolating biomass in low-oxygen conditions to inhibit decomposition, allowing the carbon fixed through photosynthesis to be stored for long durations with relatively minimal processing infrastructure and estimated costs on the order of ∼$60 tCO_2_^-1^.^39^ Other BiCRS pathways involve modest thermochemical processing. Fast-pyrolysis systems that produce bio-oil for storage avoid the costly upgrading infrastructure required for fuel production (e.g., hydrotreating and hydrogen supply), which represents a major share of capital costs in conventional thermochemical biofuel pathways.^47^ Recent techno-economic analysis of distributed fast-pyrolysis systems producing bio-oil for geologic storage further suggests relatively modest plant capital requirements and carbon removal costs of roughly ∼$83–$152 tCO_2_^-1^.^26^

In doing so, BiCRS can help establish the distributed infrastructure and regional markets needed to mobilize agricultural lignocellulosic resources at scale—bridging the current gap between forestry-based and agricultural biomass supply chains. These developments occur sequentially: BiCRS activates early markets and logistics for agricultural residues, and only later do these same assets become valuable inputs to SAF production as technologies mature.

In addition to supply-chain development, several BiCRS pathways share upstream conversion technologies with biofuels. Processes such as drying, torrefaction, and pyrolysis—used for producing stable carbon-rich intermediates for BiCRS—also serve as the front end for producing liquid intermediates that can be upgraded to transportation fuels. For example, near-term bio-oil injection for carbon storage employs modular pyrolysis systems that can later be repurposed or expanded for bio-oil upgrading to SAF once catalytic conversion technologies and policy incentives mature. This continuity in equipment, workforce, and technical expertise allows early BiCRS deployment to lower the capital and operational costs of future biorefineries. Again, these synergies materialize over time rather than concurrently; the economic benefits to SAF may emerge only after BiCRS infrastructure and experience accumulate to a critical threshold.

Among emerging BiCRS pathways, bio-oil injection represents a particularly promising near-term strategy that complements and supports longer-term bioenergy and biofuel development. Rather than being integrated directly with biofuel production within the same facility, bio-oil injection can serve as an enabling step that builds the feedstock supply chains, conversion infrastructure, and technical expertise needed for future bio-oil upgrading to SAF as technologies and policy frameworks mature. In this framework, bio-oil injection functions as a transitional phase: bio-oil produced via fast pyrolysis can be injected into deep geologic formations for durable carbon sequestration in the near term, while supporting the development of distributed biomass conversion capacity. Over time, these same systems can be redirected toward SAF production once catalytic upgrading routes such as hydrotreating or hydrocracking become more cost-effective. Unlike conventional CO_2_ sequestration, which employs high-pressure class VI wells for supercritical gas injection under the EPA Underground Injection Control program, bio-oil injection involves the emplacement of a dense, viscous, non-buoyant liquid that is immiscible with water and remains stable within confined formations at lower pressures. Class I non-hazardous industrial waste wells are well suited for dense liquid injection and entail lower infrastructure complexity and permitting costs. This distinction makes bio-oil injection a flexible and lower-capital pathway for achieving near-term carbon removal, particularly in regions lacking centralized carbon capture and storage (CCS) networks. In addition, a near-term operational synergy exists between BiCRS and bioenergy: while bio-oil can be injected for sequestration, the co-produced biochar can be combusted to provide process heat and power, enhancing overall energy efficiency and offsetting fossil fuel use. Together, these features position bio-oil injection as a practical, scalable, and complementary precursor to large-scale biofuel deployment and as an enabling step in the broader bioeconomy transition.^21^^,^^38^^,^^48^

Viewed together, BiCRS and SAF need not be interpreted as competing uses of biomass, but rather as potentially sequential components of a broader bioeconomy development pathway. In this framing, near-term BiCRS deployment can mobilize feedstocks, establish logistical networks, and generate operational experience that reduce uncertainty and investment risk for future lignocellulosic fuel facilities. This argument does not assume or model specific deployment timelines; rather, it highlights how differences in capital intensity, modularity, and market drivers may influence the pace at which supply chains mature. To the extent that early BiCRS projects help transform theoretical biomass availability into reliable, financeable, and contractible feedstock supply, they may improve the likelihood that SAF facilities can be deployed when supportive fuel and policy conditions emerge.

## A risk-minimizing approach to market, policy, and technology uncertainties

Figure 2 illustrates how uncertainty in future fuel and carbon markets—particularly SAF and carbon abatement prices—can strongly influence the economic valuation of biomass. At the time of writing, ongoing shifts in US energy policy and demand—driven by both evolving federal priorities and accelerating energy use from artificial intelligence and other technologies—illustrate the challenges of making large-scale energy investments in uncertain markets. Depending on how future policy and market scenarios are balanced, the best biomass use could be the result of synergies between biofuels and BiCRS. Recent executive and agency actions—such as EPA’s 2026–2027 Renewable Fuel Standard proposal and partial waiver of 2025 cellulosic volumes—illustrate the fluidity of incentives facing biofuel producers. In addition to policy and market uncertainty, future technological, and economic conditions are unknown. These combined uncertainty risks underscore the importance of flexible supply chains that can adapt to future conditions. Given the challenges of developing large-scale end uses in the absence of established supply chains, flexible biomass supply chains that can adapt to changing market and policy conditions—including pivoting between fuel and carbon-storage markets—can help maintain project viability.

Another major constraint on bioeconomy development is the high capital intensity and infrastructure requirements associated with conventional biofuel and bioenergy with carbon capture and storage (BECCS) pathways. Commercial-scale biofuels facilities typically require capital investments on the order of $200–$500 million to achieve economies of scale (e.g., 1,000–3,000 dry tons day^−1^ processing capacity), and the levelized cost of fuel production is highly sensitive to plant size.^19^^,^^28^^,^^49^^,^^50^ In contrast, many BiCRS applications require substantially lower capital investment and can operate viably at tens to hundreds of tons per day.^25^ BiCRS applications that have lower capital and infrastructure requirements can be deployed at smaller scales and with minimally developed or episodic biomass supply chains while providing carbon management benefits.^38^ As these supply chains for BiCRS mature, they can support a broader range of bioeconomy pathways, including biofuels. Proposed attributes of successful biomass supply chains and strategies for supply-side and demand-side growth are summarized in Table 2. These strategies align with current US efforts to streamline project approvals and expand domestic biomass utilization under EOs 14225 and 14260.

**Table 2:** Key attributes of successful biomass supply chains and proposed strategies for supply-side and demand-side bioeconomy growth

Attributes of successful bioeconomy supply chains	Strategies for bioeconomy growth
• Incrementally scalable • Adaptable to multiple uses • Optimizable through fractionation (i.e., separation into different biomass components) for value maximization • Compatible with existing infrastructure (e.g., drop-in liquid fuels) • Market driven and economically self-sustainable • Reliable	• Expand supply chains for feedstock-flexible conversion technologies • Deploy smaller-scale BiCRS pathways, such as biomass burial, to reduce risk and establish supply chains • Leverage near-term market opportunities, such as co-firing for heat and power, erosion control products, and animal bedding • Develop BiCRS pathways that produce intermediate products adaptable to future biofuels, e.g., bio-oil • Deploy carbon capture and storage opportunities across biofuel supply chains

Externalities or trade-offs of different biomass uses and supply chain configurations warrant further exploration. State and local water quality regulations may incentivize perennial crop integration in eutrophic agricultural watersheds. Demand for soil nutrients may favor distributed preprocessing for nutrient recycling. Biomass resources that are episodic (e.g., storm debris) or off-spec for biofuels conversion (e.g., high-ash material) may be better suited for BiCRS, and may provide an economic synergy between SAF and BiCRS. Changes in land-use practices for biomass production can have a positive or negative impact on carbon and environmental services. Emerging BiCRS applications, such as injecting woody biomass into shallow geologic formations, offer co-benefits like raising ground elevations to mitigate coastal flooding and can align biomass supply chains with future biofuel production needs ^e.g.,.^^48^ More mature BiCRS pathways like BECCS, can enable carbon capture at biorefineries. A mature biofuels pathway envisions converting cellulosic biomass to liquid hydrocarbons with massive external hydrogen and heat, which can convert more than 90% of the carbon to liquid fuels.^51^ External hydrogen and heat can be provided by nuclear, wind/solar and natural gas to hydrogen with sequestration of the byproduct CO_2_. A holistic supply chain-wide perspective is needed in enhancing the complementarity of a range of biomass uses.

## Summary

Biomass resources are vital for achieving energy and environmental goals, including renewable liquid biofuels for hard-to-electrify sectors and net CO_2_ emission reductions through BiCRS. However, challenges such as underdeveloped supply chains and market uncertainties hinder progress. To address these challenges, establishing flexible supply chains that minimize risks and support diverse bioeconomy pathways is essential. Aligning bioeconomy development with prevailing priorities—such as energy system reliability, sustainable land and forest management, and rural economic resilience—can enhance the long-term durability of both SAF and BiCRS pathways. These objectives are broadly consistent with international efforts to strengthen energy security while promoting responsible resource stewardship.

A coordinated strategy that emphasizes low-risk, small-scale BiCRS applications funded through existing corporate commitments for high-integrity decarbonization can lay the groundwork for robust supply chains, enabling future growth in SAF and other bioeconomy sectors. Near-term feasible actions include deployment of modular BiCRS systems under existing voluntary carbon market structures, expansion of feedstock-flexible preprocessing and aggregation infrastructure, and strengthening of MRV systems to ensure durable carbon removal. These steps can proceed under current market conditions without requiring clarity of long-term fuel price uncertainty, while preserving optionality for future large-scale fuel production. The inherent diversity of biomass resources and continuum of potential end uses for a range of products and services calls for continued research and innovation to optimize pathways, evaluate trade-offs, and ensure alignment with evolving market, policy, and technology landscapes.

## Data and code availability

All data are available in the main text or the supplementary materials.

## Acknowledgments

We gratefully acknowledge Dr. Jesse Daystar and Steven Pires of Cotton Incorporated for their valuable guidance in shaping the scope of work and for their insights in interpreting the results. This material is based upon work supported by 10.13039/100006481Cotton Incorporated under award number 25-461, and by the 10.13039/100014456Center for Bioenergy Innovation, US Department of Energy, Office of Science, Biological and Environmental Research Program under award number ERKP886.

This manuscript has been authored by UT-Battelle, LLC, under contract DE-AC05-00OR22725 with the US Department of Energy (DOE). The US government retains and the publisher, by accepting the article for publication, acknowledges that the US government retains a nonexclusive, paid-up, irrevocable, worldwide license to publish or reproduce the published form of this manuscript, or allow others to do so, for US government purposes. DOE will provide public access to these results of federally sponsored research in accordance with the DOE Public Access Plan (https://www.energy.gov/doe-public-access-plan).

Data and conversion factors used in this analysis draw in part on technical guidance provided by the National Renewable Energy Laboratory (NREL), including publicly available conversion factors documented in https://www.osti.gov/biblio/2202642.

## Author contributions

Conceptualization, M.L. and W.J.S.; methodology, C.L., W.J.S., J.F., and M.L.; investigation, M.L., C.L., and W.J.S.; visualization, M.L., C.L., W.J.S., and O.O.; funding acquisition, M.L. and W.J.S.; project administration, M.L. and W.J.S.; supervision, M.L. and W.J.S.; writing – original draft, M.L., W.J.S., C.L., J.F., and P.C.; writing – review and editing, M.L., W.J.S., C.L., J.F., D.L.S., P.C., L.M., D.d.l.T.U., R.J., N.Z., E.A.H., and C.F.

## Declaration of interests

D.L.S. provides science advisory services to Carbon Direct, Inc., Graphyte, Arbor Energy, and Earth Foundries. Other authors declare that they have no competing interests.

## Supplemental information

Document S1. Tables S1–S7, Supplementary Materials, Supplementary Text, and Equation S1–S3
