# Supplementary material for: Biomass carbon removal can help sustainable aviation fuels achieve on-time arrival
Source: iScience. 2026 May 22;29(6):115956. doi: 10.1016/j.isci.2026.115956 (PMC13224039; doi:10.1016/j.isci.2026.115956)
Supplement: Document S1. Tables S1–S7, Supplementary Materials, Supplementary Text, and Equation S1–S3 [file mmc1.pdf]

iScience, Volume *29*

Supplemental information

Biomass carbon removal can help sustainable aviation fuels achieve on-time arrival

Matthew Langholtz, Charlotte Levy, John Field, Daniel L. Sanchez, Pete Christensen, Lawrence Murdoch, Daniel de la Torre Ugarte, Oluwafemi Oyedeji, Ryan Jacobson, Ning Zeng, Emily A. Heaton, Charles Forsberg, and William Joe Sagues

Supplementary Materials for

Biomass Carbon Removal Can Help Sustainable Aviation Fuels Achieve On-Time Arrival

Authors: Matthew Langholtz, Charlotte Levy, John Field, Daniel L. Sanchez, Pete Christensen, Lawrence Murdoch, Daniel de la Torre Ugarte, Oluwafemi Oyedeji, Ryan Jacobson, Ning Zeng, Damon Hartley, Emily A. Heaton, Charles Forsberg, William Joe Sagues*

Corresponding author: wjsagues@ncsu.edu

Methods

System Boundaries and Scenario Assumptions

This analysis adopts the United States as the spatial boundary for quantitative illustrations in Figure 1. Biomass availability estimates are derived from the 2023 Billion-Ton Report (BT23)[1] and reflect supply-side production potential under near-term and mature-market conditions. These estimates incorporate environmental and economic sustainability constraints embedded in BT23, including protection of food, feed, and fiber markets, maintenance of soil and water quality, and limits on land-use change, as detailed in that report. The scenarios used here are policy- and end-use-agnostic and represent environmental and economic potential under assumed sustained market pull, rather than forecasts of realized deployment. Market price ranges used in Figure 2 are spatially agnostic and illustrative, intended to explore comparative valuation across a range of generalized fuel and carbon price conditions rather than specific policy commitments.

Figure 1, left panel

Because different biomass resources have different conversion pathways, each with different biofuels conversion efficiencies and product distributions (i.e., sustainable aviation fuels (SAF), diesel, and gasoline), a series of transformations are provided to illustrate potential growth in US SAF and biofuels production capacity in Figure 1. US biomass production capacities for the four scenarios are derived from the 2023 Billion-Ton Report (BT23), Table ES-1 [1]. Only additional biomass in the US net of current uses, and excluding landfill gas and algae is included, i.e. 317, 716, 813, and 1,051 million tonnes (Mt) per year, under the near-term, and mature-market low, medium, and high scenarios, respectively. Biomass production capacity by resource classification, scenario, and conversion pathway are provided in Table S1. These same biomass resources values are summarized by conversion pathway and scenario in Table S2. Conversion efficiencies by pathway are provided in Table S3. Biomass resources by pathway in Table S2 are multiplied by conversion efficiencies and product distribution in Table S3 to provide SAF, biodiesel, and bio-derived gasoline volumes in Table S4, which provides the values used in Figure 1, left panel.

Figure 1, right panel

A series of calculations is provided to assess the CO_2 abatement potential of biofuels and the CO_2 sequestration potential of BiCRS illustrated in Figure 1, right panel. Product-specific CO_2 emissions are provided in Table S5. These emissions factors are multiplied by the scenario- and product-specific biofuels production volumes in Table S4, under a midpoint abatement efficiency factor of 65% as a midpoint between 50% (minimum standard) and 80% (optimistic) following assumptions of DOE (2024)[2] to provide the CO_2 abatement potential of SAF and co-products in Table S6, corresponding with the values in Figure 1 for CO_2 abatement of biofuels. To assess the CO_2 removal potential of BiCRS, biomass production capacities of Table S1 are multiplied by 1.468 (assuming 50% carbon content, 3.67 units CO_2 per unit C, and a BiCRS carbon removal efficiency of 80%, following assumptions in [3]). Resulting CO_2 removal potential of BiCRS, are provided in Table S7, in billion tonnes per year. The values in Table S7 correspond with the BiCRS CO_2 removal values illustrated in Figure 1.

Table S1.

US biomass production capacity (million metric tonnes (Mt) per year, dry weight equivalent) by scenario and classification [1] and assumed conversion process [4].

			**Near-term**	**Mature market (low)**	**Mature market (med)**	**Mature market (high)**
**BT23 Resource Class**	**BT23 Resource Subclass**	**Conversion pathway**	(Mt per year, dry weight equivalent)			
Waste	FOG[1]	HEFA[2]	3	4	4	4
Waste	Other solid waste	Gasification + FT[3]	22	34	34	34
Waste	Other wet waste	Wet waste HTL[4]	29	39	39	39
Waste	Paper	Gasification + FT	58	76	76	76
Waste	Plastic	Gasification + FT	37	44	44	44
Forestry	Forest processing waste	Gasification+syngas fermentation+ATJ[5]	1	1	1	1
Forestry	Logging residues	Gasification+syngas fermentation+ATJ	17	17	17	17
Forestry	Other forest waste	Gasification+syngas fermentation+ATJ	7	7	7	7
Forestry	Small-diameter trees	Gasification+syngas fermentation+ATJ	3	32	32	32
Agriculture	Agricultural processing waste	Biochemical+ATJ	5	5	5	5
Agriculture	Agricultural residues	Biochemical+ATJ	122	143	166	186
Agriculture	Energy crops, herbaceous	Biochemical+ATJ	0	258	313	485

Agriculture	Energy crops, woody	Gasification+syngas fermentation+ATJ	0	31	48	93
Agriculture	Intermediate oilseeds	HEFA	0	25	25	25
		**Total**	**304**	**718**	**813**	**1,051**

[1]Fats, oils, and greases.
[2]Hydroprocessed esters and fatty acids.
[3]Fischer Tropsch.
[4]Hydrothermal liquefaction.
[5]Alcohol to jet.

Table S2.
Summary of Table S1 by conversion pathway.

	Near-term	Mature market (low)	Mature market (med)	Mature market (high)
**Pathway**	(Mt per year, dry weight equivalent)			
Biochemical+ATJ	127	406	484	677
Gasification + FT	117	155	155	155
Gasification+syngas fermentation+ATJ	28	88	105	151
HEFA	3	29	29	29
Wet waste HTL	29	39	39	39
**Total**	**304**	**718**	**813**	**1,051**

Table S3.

Conversion efficiency and product distribution by pathway. Conversion efficiencies for fuels are derived from Brown and Ling (2023) [4] and Charlton et al. (2025) [5]. Charlton et. al. estimate cellulosic biomass to hydrocarbon liquid fuel yields assuming 100% of the carbon is converted using massive quantities of external hydrogen. See Supplementary Text for details on commercialization and conversion rates using external hydrogen additions.

**Pathway**	**Liters of gasoline equivalent per t biomass**	**% SAF**	**% Diesel**	**% Gasoline**
Biochemical+ATJ[a]	193	90%	10%	0%
Gasification + FT[a]	246	70%	15%	15%
Gasification+syngas fermentation+ATJ[a]	271	90%	10%	0%
HEFA[a]	1,060	70%	5%	25%
Wet waste HTL[a]	446	30%	47%	23%
FT + External hydrogen[b]	690	100%	0%	0%

[a] Brown and Ling (2023).

[b] Charlton et al. (2025).

Table S4.

Calculated SAF and other biofuels production capacity by pathway and scenario. Product subclass totals correspond with values in Figure 1, left panel.

**Fuel**	**Pathway**	**Near-term**	**Mature market (low)**	**Mature market (med)**	**Mature market (high)**
		(billion liters per year)			
SAF	Biochemical+ATJ	22	71	84	118
SAF	Gasification + FT	20	27	27	27
SAF	Gasification+syngas fermentation+ATJ	7	21	26	37
SAF	HEFA	2	22	22	22
SAF	Wet waste HTL	4	5	5	5
SAF Subtotal		*55*	*146*	*163*	*208*
Diesel	Biochemical+ATJ	2	8	9	13
Diesel	Gasification + FT	4	6	6	6
Diesel	Gasification+syngas fermentation+ATJ	1	2	3	4
Diesel	HEFA	0	2	2	2
Diesel	Wet waste HTL	6	8	8	8
Diesel Subtotal		*14*	*26*	*28*	*33*
Gasoline	Biochemical+ATJ	0	0	0	0
Gasoline	Gasification + FT	4	6	6	6
Gasoline	Gasification+syngas fermentation+ATJ	0	0	0	0
Gasoline	HEFA	1	8	8	8
Gasoline	Wet waste HTL	3	4	4	4
Gasoline Subtotal		*8*	*17*	*17*	*17*
**Grand Total**		**77**	**189**	**209**	**258**

Table S5.

Table S5: CO_2 emissions coefficients by fuel type based on data from US Environmental Protection Agency [6].

**Fuel**	**CO_2 emissions (Kg CO_2 per liter fuel)**
Jet fuel	2.6
Diesel	2.7
Gasoline (Motor)	2.5

Table S6.

Simulated CO_2 abatement potential (billion tonnes (t) CO_2) by US biofuels (assumes 65% abatement efficiency, midpoint between 50% (minimum standard) and 80% (optimistic) following assumptions of DOE (2024) [2]). Results correspond with Figure 1, right panel, abatement potential of biofuels.

	**Near-term**	**Mature market (low)**	**Mature market (med)**	**Mature market (high)**
	(billion t CO_2 per year)			
SAF	0.09	0.24	0.27	0.35
Diesel	0.02	0.04	0.05	0.06
Gasoline (Motor)	0.01	0.03	0.03	0.03
**Total**	**0.13**	**0.32**	**0.35**	**0.43**

Table S7.
Simulated CO_2 sequestration potential (billion metric tonnes (t) per year) by BiCRS, assuming 50% carbon content of biomass, 3.67 units CO_2 per unit C, and a BiCRS carbon removal efficiency of 80%, following assumptions in [3].

	**Near-term**	**Mature market (low)**	**Mature market (med)**	**Mature market (high)**
	(billion t CO_2 sequestration per year)			
Wastes	0.22	0.29	0.29	0.29
Forestry	0.04	0.08	0.08	0.08
Agriculture - residues and wastes	0.19	0.68	0.82	1.17
**Total**	**0.45**	**1.05**	**1.19**	**1.54**

Assumptions and Calculations for Figure 2

Figure 2 illustrates the potential value per tonne of cellulosic biomass that could be realized under a range of market prices for fuels and carbon abatement under generalized SAF and BiCRS. Future conversion efficiencies, costs, and prices for SAF and BiCRS are unknown, and assumptions here are not provided as definitive values. A sensitivity analysis around prices is provided in Figure 2 to illustrate the range of comparative advantages under a range of future market scenarios.

Values per tonne of biomass from biofuels (e.g. SAF) are calculated as illustrated in Equation 1:

Equation 1:

$$\frac{liters\ biofuel}{tonne\ biomass} * \left(\frac{price}{liter} - \frac{cost}{liter}\right) = \frac{value}{tonne\ biomass}$$

Where;
liters = assumed conversion yield of biofuels (e.g. SAF)
price = assumed market price for biofuels (e.g. SAF)
cost = assumed break-even cost of production for biofuels, including cost of capital, but not profit (e.g. SAF)
Conversion yield is assumed to be 250 liters per tonne of biomass (corresponding to 60 gallons per short ton of biomass[7], within the range of 45-65 gallons per ton for FT + gasification or pyrolysis[8,9]).
Cost of production is assumed $1 per liter. This is within the range of values reported by Bann et al (2017)[10] and is used as an optimistic mature-market cost, though pioneer costs are expected to be higher (Wang et al. 2024)[11].
Biofuel market prices are simulated with a sensitivity analysis provided in Figure 2.

Values per tonne of biomass from carbon abatement are calculated as illustrated in Equation 2:

Equation 2:

$$\frac{tonnes\ CO_2\ abated_{(i,j)}}{tonne\ biomass} * \frac{price - cost_{(i,j)}}{tonne\ CO_2} = \frac{value}{tonne\ biomass}$$

Where:
i and *j* represent values for SAF and BiCRS, respectively
tonnes CO_2 abated = tonnes of CO_2 avoided by SAF or removed by BiCRS per tonne of biomass
price=assumed market price for carbon abatement
cost=assumed break-even cost of carbon abatement (defined in Equation 3).
Abatement efficiencies are assumed to be 0.7 (i.e., carbon conversion efficiency of 0.20 unit C-fuel per unit biomass[7] * 3.67 units CO_2 per unit of C) and 1.6 tonnes (i.e., carbon conversion efficiency of 0.45 unit C-removed per unit biomass * 3.67 units CO2 per unit of C, comparable to Sanchez et al (2025)[12], Yablanovich and Deckman (2023)[13], and Chapter 3, Table 3, in IPCC (2019)[14]) CO_2 per tonne of biomass for SAF and BiCRS, respectively.
Costs of carbon abatement are assumed to be $200 and $50 per tonne CO_2 for SAF [2] and BiCRS [13,15,16], respectively, and these values are intentionally optimistic for both pathways to reflect best-case, lower-bound abatement costs

Carbon abatement market prices are simulated with a sensitivity analysis provided in Figure 2. In instances where costs exceed prices, we assume valuations are zero rather than negative, though this future analyses could alternatively assume negative values. As described above, carbon abatement costs in this analysis are referenced from other sources and are not calculated in this analysis. However, the calculation for carbon abatement cost is provided in Equation 3 for reference.

Equation 3:

$$\frac{price_{abatement} - price_{conventional}}{E_{conventional} - E_{abatement}} = \frac{CAC}{tonne\ CO_2}$$

Where:
CAC=carbon abatement cost
$price_{abatement}$ = unit price of abatement strategy
$price_{conventional}$=unit price of the conventional product
$E_{conventional}$=unit emissions of the conventional product
$E_{abatement}$=unit emissions of the abatement strategy

Supplementary Text

Additional supporting documentation

Additional documentation is provided to complement references used in the manuscript.

- Acknowledgement of challenges of SAF deployment as of 2024 are presented by the International Air Transportation Association [17].
- Carbon vaulting through wood burial as an efficient carbon dioxide removal (CDR) strategy is presented by Neng et al. (2008, 2024) [16,18].
- Exploring the carbon efficiency and energy use of a range of CDR strategies, Sanchez et al (2025) [12] identify biomass-based CDR approaches as efficient due to high carbon contents and low energy requirements.
- The potential magnitude and durability of BECCS and BiCRS pathways is explored in Dees et al. (2023) [19], finding that non-BECCS BiCRS pathways can provide roughly half the carbon draw-down magnitude of BECCS, and retaining more than half their initial drawdown over 100 years.
- Developer scale-up plans for BiCRS are provided in a recent report by Carbon Direct [20].
- In addition to the carbon abatement costs provided in the text, Woodall and McCormick (2022) [21] demonstrate the Aines Principle, where the value of carbon in biomass can exceed it the value of its energy content, under reference values for energy vs carbon benefits.
- Expanding on discussion of external hydrogen inputs in Table S3, DG Fuels has announced plans to build several cellulosic biomass to jet fuel plants with massive external hydrogen additions to minimize feedstock. Each plant will cost $ 5 billion using flowsheets developed by Honeywell UOP. These market-driven flowsheets show 93% of all carbon is converted to jet fuel equivalent. The flowsheet is

designed to maximize jet fuel rather than a broad distribution of hydrocarbon products because of financial incentives for SAF.

References

1. USDOE (2024). 2023 Billion-Ton Report: An Assessment of U.S. Renewable Carbon Resources (Oak Ridge National Laboratory).

2. DOE (2024). Pathways to Commercial Liftoff: Sustainable Aviation Fuel.

3. Pett-Ridge, J., Kuebbing, S., Mayer, A., Hovorka, S., Pilorgé, H., Baker, S., Pang, S., Scown, C., Mayfield, K., Wong, A., et al. (2023). Roads to Removal: Options for Carbon Dioxide Removal in the United States https://doi.org/10.2172/2301853.

4. Brown, C., and Tao, L. (2023). Biofuel Production and Greenhouse Gas Reduction Potential (National Renewable Energy Laboratory (NREL), Golden, CO (United States)) https://doi.org/10.2172/2202642.

5. Charlton, T.W., Forsberg, C.W., and Dale, B.E. (2025). Potential U.S. Production of Liquid Hydrocarbons From Biomass With Addition of Massive External Heat and Hydrogen Inputs. GCB Bioenergy *17*, e70022. https://doi.org/10.1111/gcbb.70022.

6. U.S. Energy Information Administration EIA - Independent Statistics and Analysis. Carbon Dioxide Emiss. Coeff. https://www.eia.gov/environment/emissions/co2_vol_mass.php.

7. Rosales Calderon, O., Tao, L., Abdullah, Z., Moriarty, K., Smolinski, S., Milbrandt, A., Talmadge, M., Bhatt, A., Zhang, Y., Ravi, V., et al. (2024). Sustainable Aviation Fuel (SAF) State-of-Industry Report: State of SAF Production Process https://doi.org/10.2172/2426562.

8. Wright, M.M., Daugaard, D.E., Satrio, J.A., and Brown, R.C. (2010). Techno-economic analysis of biomass fast pyrolysis to transportation fuels. Fuel *89*, S2–S10. https://doi.org/10.1016/j.fuel.2010.07.029.

9. Davis, R., Tao, L., Scarlata, C., Tan, E.C.D., Ross, J., Lukas, J., and Sexton, D. (2015). Process Design and Economics for the Conversion of Lignocellulosic Biomass to Hydrocarbons: Dilute-Acid and Enzymatic Deconstruction of Biomass to Sugars and Catalytic Conversion of Sugars to Hydrocarbons https://doi.org/10.2172/1176746.

10. Bann, S.J., Malina, R., Staples, M.D., Suresh, P., Pearlson, M., Tyner, W.E., Hileman, J.I., and Barrett, S. (2017). The costs of production of alternative jet fuel: A harmonized stochastic assessment. Bioresour. Technol. *227*, 179–187. https://doi.org/10.1016/j.biortech.2016.12.032.

11. Wang, B., Ting, Z.J., and Zhao, M. (2024). Sustainable aviation fuels: Key opportunities and challenges in lowering carbon emissions for aviation industry. Carbon Capture Sci. Technol. *13*, 100263. https://doi.org/10.1016/j.ccst.2024.100263.

12. Sanchez, D.L., Psarras, P., Murnen, H.K., and Rogers, B. (2025). Carbon removal efficiency and energy requirement of engineered carbon removal technologies. RSC Sustain. https://doi.org/10.1039/D4SU00552J.

13. Yablonovitch, E., and Deckman, H.W. (2023). Scalable, economical, and stable sequestration of agricultural fixed carbon. Proc. Natl. Acad. Sci. *120*, e2217695120. https://doi.org/10.1073/pnas.2217695120.

14. 2019 Refinement to the 2006 IPCC Guidelines for National Greenhouse Gas Inventories — IPCC https://www.ipcc.ch/report/2019-refinement-to-the-2006-ipcc-guidelines-for-national-greenhouse-gas-inventories/.

15. Zeng, N., and Hausmann, H. (2022). Wood Vault: remove atmospheric CO2 with trees, store wood for carbon sequestration for now and as biomass, bioenergy and carbon reserve for the future. Carbon Balance Manag. *17*, 2. https://doi.org/10.1186/s13021-022-00202-0.

16. Zeng, N., Zhao, X., Poisson, G., Clifford, B., Liu, Y., Liu, H., Meng, T., Picard, L., Zeng-Mariotti, E., Zaitchik, B., et al. (2024). 3775-year-old wood burial supports “wood vaulting” as a durable carbon removal method. Science *385*, 1454–1459. https://doi.org/10.1126/science.adm8133.

17. International Air Transport Association (2024). Disappointingly Slow Growth in SAF Production. https://www.iata.org/en/pressroom/2024-releases/2024-12-10-03/.

18. Zeng, N. (2008). Carbon sequestration via wood burial. Carbon Balance Manag. *3*, 1. https://doi.org/10.1186/1750-0680-3-1.

19. Dees, J.P., Sagues, W.J., Woods, E., Goldstein, H.M., Simon, A.J., and Sanchez, D.L. (2023). Leveraging the bioeconomy for carbon drawdown. Green Chem. *25*, 2930–2957. https://doi.org/10.1039/D2GC02483G.

20. Cabiyo, B., Stielglitz-Courtney, M., Garcia, D.M., Oalican, N., Atreyea, M., Goldberg, J., Macfarlane, M., O’Connor-Morberg, S., Potts, M., Sobieski, M., et al. (2024). 2024 State of the Voluntary Carbon Market Report (Carbon Direct).

21. Woodall, C.M., and McCormick, C.F. (2022). Assessing the optimal uses of biomass: Carbon and energy price conditions for the Aines Principle to apply. Front. Clim. *4*. https://doi.org/10.3389/fclim.2022.993230.
